# Study on the stability analysis of rainfall slope based on G-A model considering moisture content

**DOI:** 10.1038/s41598-022-14628-6

**Published:** 2022-06-21

**Authors:** Li Li, Dongsheng Zhao, Bo Ni, Yue Qiang, Gang Liu, Ling Zhou

**Affiliations:** School of Civil Engineering, Chongqing Three Gorges University, WanzhouChongqing, 404100 China

**Keywords:** Natural hazards, Hydrogeology

## Abstract

In this paper, the moisture content on the wetting front is obtained by using the Van Genuchten (VG) model of unsaturated soil, and then the distribution of moisture content in the upper part of the wetting front is simplified as a trapezoid. The Green-Ampt (G-A) infiltration model of infinite slope with unsaturated characteristics is derived. The analytical expression of safety coefficient (*FOS*) of infinite slope with rainfall is solved by combining the limit equilibrium method with the unsaturated soil shear strength theory phase. The results show that: 1) compared with the traditional G-A model and the combined rectangular and 1/4 oval model, the upper part of the wetting front is simplified to a trapezoidal model, which has great advantages in infiltration rate and cumulative infiltration, especially when the slope is large or the rain intensity is heavy; 2) since the distribution of soil moisture content above the wetting front is considered, the matrix suction at the wetting front is not neglected, and the safety coefficient calculated by the method proposed in this paper is closer to the actual situation than the traditional G-A model.

## Introduction

Landslide is the most common natural disaster. There are many factors that cause landslides, such as rainfall, earthquakes, artificial slope cutting, reservoir water level fluctuation and so on^1^. Rainfall is one of the main factors causing landslides, in the process of rainfall infiltration, the suction of slope matrix decreases gradually, the anti-sliding force of slope decreases, and the sliding force increases, which finally leads to landslide^2^. At the same time, the fluid flow will induce instability of geomaterials^3^. Therefore, it is essential to analyze the rainfall infiltration law for slope stability analysis. At present, the common rainfall infiltration models are Green-Ampt model^4^, Kostiakov model^5^, Horton model^6^, Philip model^7^ and Richards Eq.^8^.

The Green-Ampt (G-A) model is similar to Darcy’s law, which is simple, with clear physical meaning and the scalability is well^9^. It has been widely used in infiltration studies since the G-A model was proposed. At the same time, the parameters of the model are modified and supplemented, which greatly improved the scope of application of the G-A Model. The relationship between rainfall intensity and infiltration was analyzed, and the G-A model was extended to infiltration under steady rainfall condition^10^. Based on the study of Mein and Larson, the G-A was further developed by Chu^11^ to calculate soil infiltration performance during non-uniform rainfall. The influence of slope was considered, and an improved infiltration G-A model suitable for rainfall-induced slope infiltration problem was proposed by Chen, et al.^12^. At the same time, G-A models are often used in slope stability analysis, and these proposed methods are applied to landslide analysis^13,14^.

In the above study, the influence of the unsaturated interval above the wetting front on infiltration is ignored. To obtain a more accurate infiltration model, many scholars have carried out related studies on the distribution of moisture content in the upper part of the wetting front. The soil hydraulic conductivity after the wetting front was taken as 0.5 times the saturated hydraulic conductivity and has been successfully used in irrigation by Bouwer, et al^15^. Based on the sand infiltration experiment, wetting area were divided into saturated area and unsaturated area, and the characteristics of soil moisture slope variation were established by Wang, et al.^16^. Through infiltration experiments, it was suggested that the moisture content profile of the unsaturated wetting area is equivalent to an ellipse, and the Green-Ampt model was improved Peng, et al.^17^. Compared with the two assumptions that the water content distribution above the wetting front is rectangular and 1/4 ellipse combined model and traditional G-A model, Zhang, et al.^18^ found that the trapezoidal moisture content distribution is more line with the infiltration law. However, this study result only considers the horizontal infiltration situation, and has not considered the slope infiltration situation.

## Stability analysis of unsaturated soil slope

### Assumptions

Due to the complexity of realistic slope conditions, to facilitate the solution, the following assumptions are made before the theoretical derivation:

1. The rainfall is uniform (i.e., the rainfall intensity is constant);
2. The soil is homogeneous;
3. Unsaturated soil with initial moisture content below the wetting front;
4. When rainfall infiltrates, there is a prominent wetting front in the slope;
5. The slope is simplified as an infinite slope.

### Green-Ampt model wetting front depth calculation

When the rainfall intensity is less than the infiltration rate, according to the Green-Ampt infiltration model, the infiltration rate *i* in the slope determined by the rainfall intensity is expressed as follows:

1 $$ i = q_{r} \cos \alpha $$

where *i* represents infiltration rate, *q*_r_ represents rainfall intensity, and *α* represents slope angle.

The relationship between rainfall duration *t* and infiltration depth *z*_f_ can be expressed as follows:

2 $$ z_{{\text{f}}} = \frac{i}{{\theta_{{\text{s}}} - \theta_{{\text{i}}} }}t $$

where *θ*_s_ and *θ*_i_ represent the saturated moisture content and the initial moisture content.

In the traditional G-A model, the infiltration rate *i* is expressed as follows:

3 $$ i = K_{{\text{s}}} \frac{{z_{{\text{f}}} + h_{{0}} + h_{{\text{f}}} }}{{z_{{\text{f}}} }} $$

where *K*_s_ is the saturated hydraulic conductivity, *h*_0_ is the depth of ponding, *h*_f_ represents the suction head of the wetting front, and *z*_f_ represents the wetting front depth. For an infinite homogeneous slope, in the stable infiltration process, the following relations exist:

4 $$ i_{{}} dt = \left( {\theta_{{\text{s}}} - \theta_{{\text{i}}} } \right)dz_{{\text{f}}} $$

Combining Eqs. (3) and (4), the relationship between wetting front and infiltration time is as follows:

5 $$ t = \frac{{\theta_{{\text{s}}} - \theta_{{\text{i}}} }}{{K_{{\text{s}}} }}\left[ {z_{{\text{f}}} - \left( {h_{{0}} + h_{{\text{f}}} } \right)\ln \frac{{z_{{\text{f}}} + h_{{0}} + h_{{\text{f}}} }}{{h_{0} + h_{{\text{f}}} }}} \right] $$

Therefore, when the rainfall intensity is greater than the infiltration rate, in the traditional G-A model, the relationship between the depth of the wetting front *z*_f_ and the rainfall duration *t* is expressed as:

6 $$ \left\{ \begin{gathered} z_{{\text{f}}} = \frac{{q_{{\text{r}}} \cos \alpha }}{{\theta_{{\text{s}}} - \theta_{{\text{i}}} }}t,0 \le t \le t_{{\text{p}}} \hfill \\ t - t_{{\text{p}}} = \frac{{\theta_{{\text{s}}} - \theta_{{\text{i}}} }}{{K_{{\text{s}}} }}\left[ {z_{{\text{f}}} - z_{{\text{p}}} - h_{{\text{f}}} \ln \frac{{z_{{\text{f}}} + h{\text{f}}}}{{z_{{\text{p}}} + h_{{\text{f}}} }}} \right],t > t_{p} \hfill \\ \end{gathered} \right. $$

### Improved Green-A model considering moisture content

**Figure 1:**
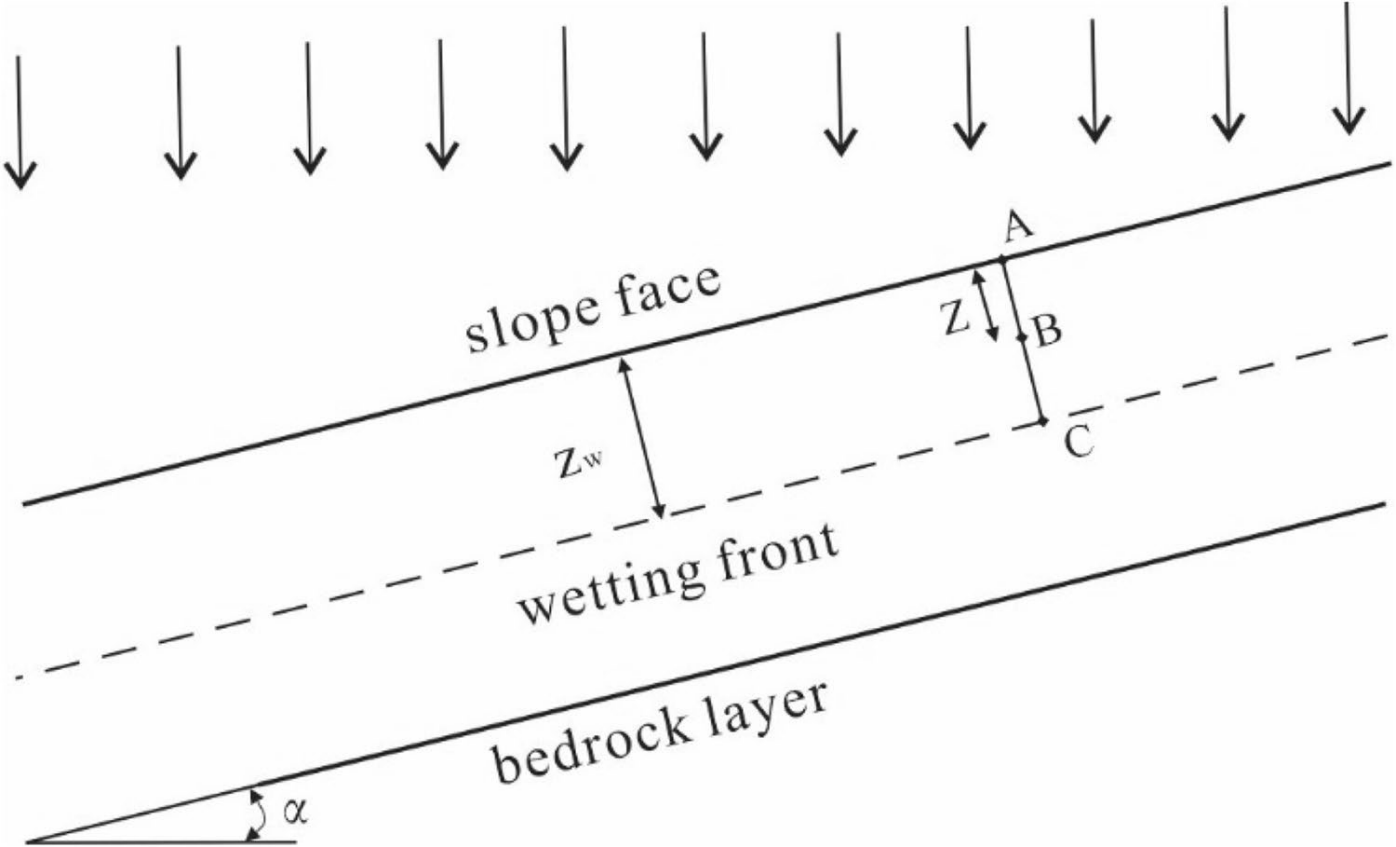
Sketch of rainfall infiltration in slope.

It is difficult to reach a fully saturated state during slope infiltration. The moisture content in the soil mainly depends on the soil matrix suction, in order to obtain the distribution of moisture content in the wetting area during the rainfall infiltration process, it is necessary to analyze the distribution of the matrix suction during the rainfall infiltration process. The schematic diagram of the calculation of rainfall-induced slope infiltration is shown in Fig. 1. The downward direction of the vertical slope is selected as the positive direction. To obtain the variation law of the matric suction in the wetting area, point A in the slope, point B in the wetting area and point C in the wetting front are selected as the calculation points. The driving force of soil moisture movement is mainly determined by the potential energy difference between the soil potential energy at a certain position and the reference object^19^. According to the potential energy equation, the total potential energy is expressed as follows:

7 $$ \psi = \psi_{g} + \psi_{p} + \psi_{m} $$

where *ψ* is the total potential energy of water in the soil; *ψ*_g_ is the gravitational potential energy; *ψ*_p_ is the pressure potential energy; *ψ*_m_ is the matrix potential energy; *h* is the matric suction head; Then the total potential energy of three points A, B and C is expressed as follows:

8 $$ \psi_{{\text{A}}} = \, 0 $$

9 $$ \psi_{{\text{B}}} = z{\text{cos}}\alpha + h_{{\text{b}}} ({\text{B}}) $$

10 $$ \psi_{{\text{C}}} = z_{{\text{f}}} {\text{cos}}\alpha + h_{{\text{c}}} ({\text{C}}) $$

According to Darcy’s law:

11 $$ i = - K\left( {\uptheta } \right)\frac{d\psi }{{dz}} $$

where *K*(θ) is an unsaturated permeability coefficient.

The infiltration rate between A and B and between B and C are respectively:

12 $$ i = - K\left( \theta \right)\frac{{\psi_{{\text{B}}} - \psi_{{\text{A}}} }}{z} $$

13 $$ i = - K\left( \theta \right)\frac{{\psi_{{\text{C}}} - \psi_{{\text{A}}} }}{{z_{{\text{f}}} }} $$

From the Eqs. (8) (9) (10) (12) and (13), it can be known that the matrix suction at point B is expressed as follows:

14 $$ h_{B} = \frac{{h_{{\text{f}}} }}{{z_{{\text{f}}} }}z $$

Matrix suction at any depth in the soil infiltration profile can be calculated from Eq. (14).

The Van Genuchten model is a model that describes the relationship between soil water-holding capacity and matrix suction, and this model has been recognized and widely used by scholars^20^. The specific expression is as follows:

15 $$ \theta (z) = \theta_{{\text{r}}} + \frac{{\theta_{{\text{s}}} - \theta_{{\text{r}}} }}{{\left[ {1 + \left( {\alpha h} \right)^{n} } \right]^{m} }} $$

where *θ*(*z*) represents the moisture content of the soil when the depth is *z*, *θ*_s_ represents the saturated moisture content of soil volume, *θ*_r_ is the residual moisture content, *h* is the pressure water head, *α n* and *m* are fitting parameters.

Combining Eqs. (14) and (15), the moisture content *θ*(*z*) at any depth in the wetting area can be obtained as:

16 $$ \theta (z) = \theta_{{\text{r}}} + \frac{{\theta_{{\text{s}}} - \theta_{{\text{r}}} }}{{\left[ {1 + \left( {\frac{z}{{z_{{\text{f}}} }}\alpha h_{{\text{f}}} } \right)^{n} } \right]^{m} }} $$

The cumulative infiltration amount *F* of rainfall can be calculated as follows:

17 $$ F = \int_{0}^{{z_{{\text{f}}} }} {\left[ {\theta \left( z \right) - \theta_{{\text{i}}} } \right]} dz $$

Combining Eqs. (16) and (17), the cumulative infiltration amount can be expressed as follows:

18 $$ F = \left( {\theta_{{\text{r}}} - \theta_{{\text{i}}} } \right)z_{{\text{f}}} + \left( {\theta_{{\text{s}}} - \theta_{{\text{r}}} } \right)\int_{0}^{{z_{{\text{f}}} }} {\frac{1}{{\left[ {1 + \left( {\frac{{\alpha h_{{\text{f}}} }}{{z_{{\text{f}}} }}z} \right)^{n} } \right]^{m} }}} dz $$

**Figure 2:**
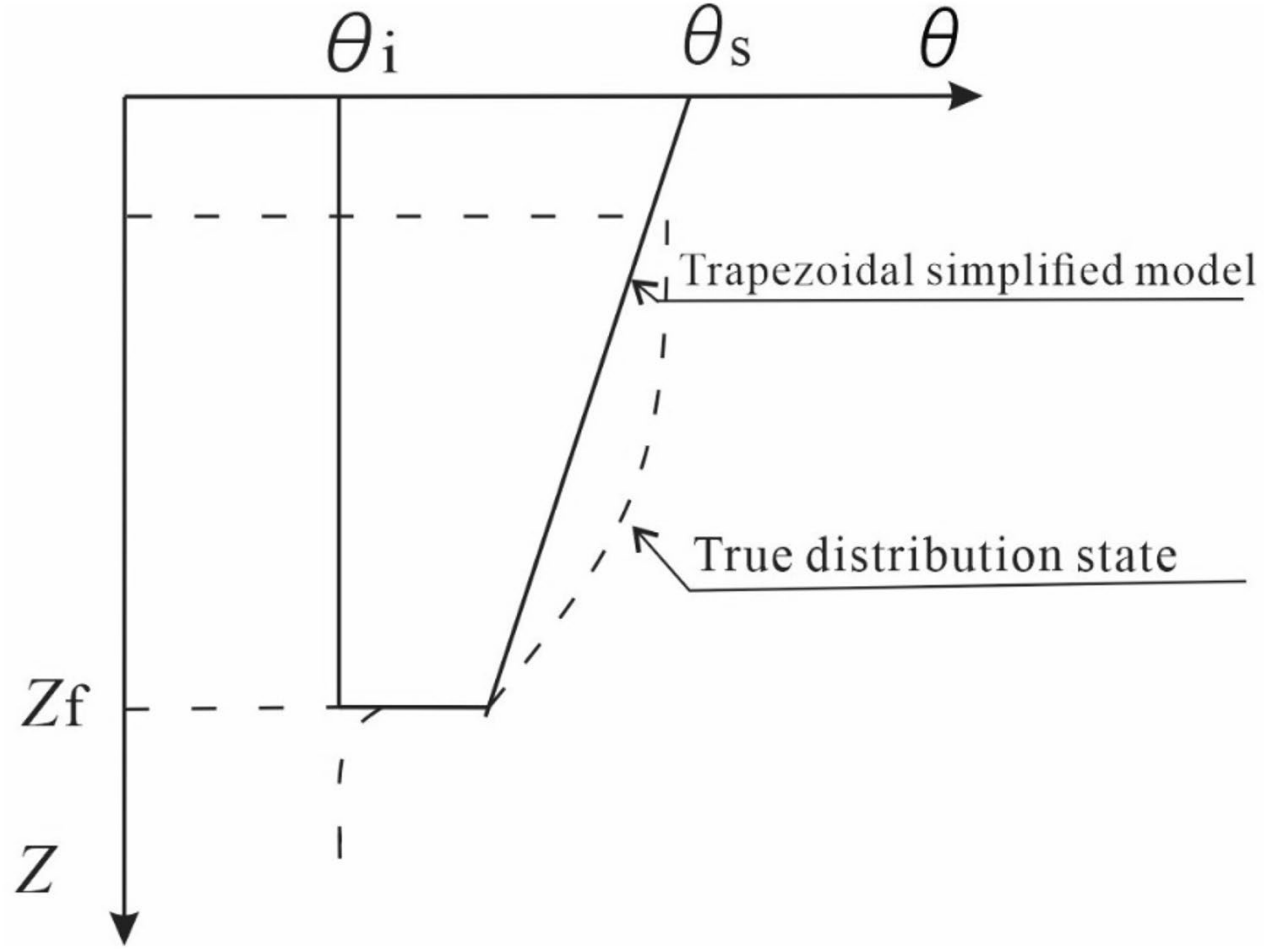
Simplified moisture content diagram of soil section.

In order to facilitate the calculation, the above equation should be simplified. According to the Eq. (18) and referring to the results of trapezoidal moisture content distribution and rectangular and 1/4 elliptical moisture content distribution proposed by Zhang, et al.^18^, the soil moisture content above of wetting front is simplified to trapezoid, as shown in Fig. 2. The moisture content distribution of the slope can be calculated as follows:

Put *z* = *z*_f_ into the Eq. (18), the moisture content (*θ*_*z*f_) of the wetting front can be obtained as follows:

19 $$ \theta_{{{\text{zf}}}} = \theta_{{\text{r}}} + \frac{{\theta_{{\text{s}}} - \theta_{{\text{r}}} }}{{\left[ {1 + \left( {\alpha h_{{\text{f}}} } \right)^{n} } \right]^{m} }} $$

Simplified moisture content in the upper of the wetting front according to trapezoid, the soil moisture content *θ*(*z*) at any depth can be simplified as:

20 $$ \theta \left( z \right) = \left\{ \begin{gathered} \theta_{{{\text{zf}}}} + \left( {\theta_{{\text{s}}} - \theta_{{{\text{zf}}}} } \right)(1 - \frac{z}{{z_{{\text{f}}} }}),z \le z_{{\text{f}}} \hfill \\ \theta_{{\text{i}}} ,z \ge z_{{\text{f}}} \hfill \\ \end{gathered} \right. $$

Combining Eqs. (17) and (20), the cumulative infiltration is expressed as follows:

21 $$ F = \left( {\theta_{{{\text{zf}}}} - \theta_{{\text{i}}} } \right)z_{{\text{f}}} + \frac{1}{2}z_{{\text{f}}} (\theta_{{\text{s}}} - \theta_{{{\text{zf}}}} ) $$

The rate of infiltration *i* can be derived from Eq. (21):

22 $$ i = \left( {\theta_{{{\text{zf}}}} - \theta_{i} } \right)\frac{{dz_{{\text{f}}} }}{dt} + \frac{1}{2}(\theta_{{\text{s}}} - \theta_{{{\text{zf}}}} )\frac{{dz_{{\text{f}}} }}{dt} $$

In the beginning of the rainfall, the rainfall intensity is less than the infiltration rate, the rainfall intensity controls the infiltration rate of the soil, and the infiltration rate *q*_w_ is:

23 $$ i = q_{{\text{w}}} = q_{{\text{r}}} \cos \alpha $$

As the rainfall progresses, the slope’s surface tends to be ponding, and the infiltration capacity of the slope gradually decreases. According to the G-A model, the infiltration rate of the slope can be expressed as:

24 $$ i = K_{{\text{s}}} \left( {\frac{{z_{{\text{f}}} \cos \alpha + h_{{\text{f}}} }}{{z_{{\text{w}}} }}} \right) $$

Due to the continuous change of infiltration rate, there is a critical time *t*_p_, and the corresponding infiltration depth is *z*_p_. At this time, the component of rainfall intensity in the direction of the slope is equal to the infiltration capacity of the soil, based on the infiltration rate, the following equation is established:

25 $$ q_{{\text{r}}} \cos \alpha = K_{{\text{s}}} \frac{{z_{{\text{p}}} \cos \alpha + h_{{\text{f}}} }}{{z_{{\text{p}}} }} $$

The infiltration depth is expressed as follows:

26 $$ z_{{\text{p}}} = K_{{\text{s}}} \frac{{h_{{\text{f}}} }}{{(q_{{\text{r}}} - K_{{\text{s}}} )\cos \alpha }} $$

Combined with Eqs. (24) and (26), the critical cumulative infiltration *I*_p_ can be obtained:

27 $$ I_{{\text{p}}} = \left( {\theta_{{{\text{zf}}}} - \theta_{{\text{i}}} } \right)z_{{\text{p}}} + \frac{1}{2}\left( {\theta_{{\text{s}}} - \theta_{{{\text{z}}_{{\text{f}}} }} } \right)z_{{\text{p}}} $$

In the joint Eqs. (27) and (23), the critical time point *t*_p_ can be expressed as follows:

28 $$ t_{{\text{p}}} = \frac{{I_{{\text{p}}} }}{{q_{{\text{r}}} \cos \alpha }} = \frac{{\left( {\theta_{{{\text{zf}}}} - \theta_{{\text{i}}} } \right)z_{{\text{p}}} + \frac{1}{2}\left( {\theta_{{\text{s}}} - \theta_{{{\text{zf}}}} } \right)z_{{\text{p}}} }}{{q_{{\text{r}}} \cos \alpha }} $$

When the rainfall intensity is less than the infiltration rate of the soil itself, that is, when *t* < *t*_p_:

29 $$ \left( {\theta_{{{\text{zf}}}} - \theta_{i} } \right)\frac{{dz_{{\text{f}}} }}{dt} + \frac{1}{2}\left( {\theta_{{\text{s}}} - \theta_{{{\text{z}}_{{\text{f}}} }} } \right)\frac{{dz_{{\text{f}}} }}{dt} = q_{{\text{r}}} \cos \alpha $$

30 $$ \frac{{dz_{{\text{f}}} }}{dt} = \frac{{q_{{\text{r}}} \cos \alpha }}{{\left( {\theta_{{{\text{zf}}}} - \theta_{i} } \right) + \frac{1}{2}\left( {\theta_{{\text{s}}} - \theta_{{{\text{zf}}}} } \right)}} $$

When the rainfall intensity is greater than the infiltration rate of the soil, that is, *t* > *t*_p_, the infiltration rate of the soil is determined by the soil itself:

31 $$ \left( {\theta_{{{\text{zf}}}} - \theta_{i} } \right)\frac{{dz_{{\text{f}}} }}{dt} + \frac{1}{2}\left( {\theta_{{\text{s}}} - \theta_{{{\text{z}}_{{\text{f}}} }} } \right)\frac{{dz_{{\text{f}}} }}{dt} = K_{{\text{s}}} \frac{{z_{{\text{f}}} \cos \alpha + h_{{\text{f}}} }}{{z_{{\text{f}}} }} $$

32 $$ \frac{{dz_{{\text{f}}} }}{dt} = \frac{{K_{{\text{s}}} (\cos \alpha + \frac{{h_{{\text{f}}} }}{{z_{{\text{f}}} }})}}{{\left( {\theta_{{{\text{zf}}}} - \theta_{{\text{i}}} } \right) + \frac{1}{2}\left( {\theta_{{\text{s}}} - \theta_{{{\text{zf}}}} } \right)}} $$

To sum up, the relationship between the development rate of the wetting front depth and the rainfall time in the whole process of rainfall infiltration is as follows:

33 $$ \frac{{dz_{{\text{f}}} }}{dt} = \left\{ \begin{gathered} \frac{{q_{{\text{r}}} \cos \alpha }}{{\left( {\theta_{{{\text{zf}}}} - \theta_{i} } \right) + \frac{1}{2}\left( {\theta_{{\text{s}}} - \theta_{{{\text{zf}}}} } \right)}},t \le t_{{\text{p}}} \hfill \\ \frac{{K_{{\text{s}}} (\cos \alpha + \frac{{h_{{\text{f}}} }}{{z_{{\text{f}}} }})}}{{\left( {\theta_{{{\text{zf}}}} - \theta_{{\text{i}}} } \right) + \frac{1}{2}\left( {\theta_{{\text{s}}} - \theta_{{{\text{zf}}}} } \right)}},t \ge t_{{\text{p}}} \hfill \\ \end{gathered} \right. $$

Integrate Eq. (33) and substitute the initial boundary conditions *t* = 0, *z*_f_ = 0, the infiltration critical conditions *t* = *t*_p_, *z*_f_ = *z*_p_, the relationship between time and infiltration depth in the process of infiltration can be obtained:

34 $$ t = \left\{ \begin{gathered} \frac{{\left( {\theta_{{{\text{zf}}}} - \theta_{{\text{i}}} } \right) + \frac{1}{2}\left( {\theta_{{\text{s}}} - \theta_{{{\text{zf}}}} } \right)}}{{q_{{\text{r}}} \cos \alpha }}z_{{\text{f}}} ,t \le t_{{\text{p}}} \hfill \\ \frac{{\left( {\theta_{{z{\text{f}}}} - \theta_{{\text{i}}} } \right) + \frac{1}{2}\left( {\theta_{{\text{s}}} - \theta_{{{\text{zf}}}} } \right)}}{{K_{{\text{s}}} }} \cdot \left[ {\left( {z_{{\text{f}}} - z_{{\text{p}}} } \right) - \frac{{h_{{\text{f}}} }}{\cos \beta }\ln \frac{{z_{{\text{f}}} \cos \beta + h_{{\text{f}}} }}{{z_{{\text{p}}} \cos \beta + h_{{\text{f}}} }}} \right] + t_{{\text{p}}} ,t \ge t_{{\text{p}}} \hfill \\ \end{gathered} \right. $$

The dynamic change with time of wetting front depth during rainfall infiltration is expressed as Eq. (34). Compared with the improved G-A model of Eq. (6). The model in this paper considers the change of moisture content in the wetting area and the depth during the rainfall infiltration process.

### Calculation of stability coefficient of infinite slope

**Figure 3:**
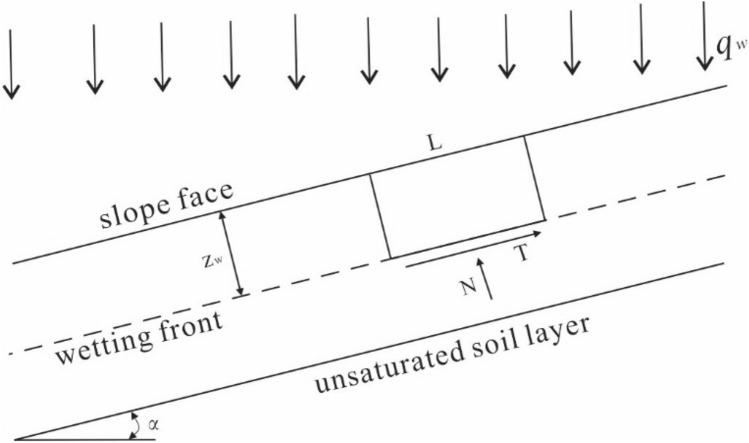
Schematic diagram of stability analysis of infinite slope under rainfall.

The slope infiltration is simplified as shown in Fig. 3 and a representative soil block is taken for force analysis. In rainfall infiltration, the soil weight changes with the change of moisture content. The weight of soil at any depth is expressed as follows:

35 $$ \gamma \left( z \right) = \left\{ \begin{gathered} \left[ {1 + \theta (z)} \right]\gamma_{{\text{d}}} \hfill \\ \gamma_{{\text{i}}} \hfill \\ \end{gathered} \right. $$

where *γ*(*z*) is the soil weight at the depth *z* of the wetting area; *γ*_*d*_ is the dry weight of the soil; *γ*_*i*_ is the initial weight of the soil. By combining Eqs. (35) and (20), the soil weight at any depth above the wetting front can be expressed as follows:

36 $$ \gamma \left( z \right) = \left\{ \begin{gathered} \left[ {1 + \theta_{{{\text{zf}}}} + \left( {\theta_{{\text{s}}} - \theta_{{{\text{zf}}}} } \right)\left( {1 - \frac{z}{{z_{{\text{f}}} }}} \right)} \right]\gamma_{{\text{d}}} \hfill \\ \gamma_{{\text{i}}} \hfill \\ \end{gathered} \right. $$

The weight *G* of the representative soil strip is expressed as follows:

37 $$ G_{{\text{w}}} = \int_{0}^{{z_{{\text{f}}} }} {\gamma \left( z \right)} Ldz = \int_{0}^{{z_{{\text{f}}} }} {\left[ {1 + \theta_{{z{\text{f}}}} + \left( {\theta_{{\text{s}}} - \theta_{{{\text{zf}}}} } \right)(1 - \frac{z}{{z_{{\text{p}}} }})} \right]} \gamma_{{\text{d}}} Ldz = \left[ {1 + \theta_{{{\text{zf}}}} + \frac{1}{2}\left( {\theta_{{\text{s}}} - \theta_{{z{\text{f}}}} } \right)} \right]\gamma_{{\text{d}}} Ldz $$

Taking the area of *L* × 1 for analysis, the sliding surface shear stress *τ*_*s*_ and normal stress *σ* can be obtained from the mechanical equilibrium equation as:

38 $$ \tau_{{\text{s}}} = \left[ {1 + \theta_{{{\text{zf}}}} + \frac{1}{2}\left( {\theta_{{\text{s}}} - \theta_{{{\text{zf}}}} } \right)} \right]\gamma_{{\text{d}}} z_{{\text{f}}} \sin \alpha $$

39 $$ \sigma_{{\text{s}}} = \left[ {1 + \theta_{{{\text{zf}}}} + \frac{1}{2}\left( {\theta_{{\text{s}}} - \theta_{{{\text{zf}}}} } \right)} \right]\gamma_{{\text{d}}} z_{{\text{f}}} \cos \alpha $$

Based on the unified effective stress principle of unsaturated soil^21^, the effective stress is expressed as follows:

40 $$ \sigma^{^{\prime}} = \sigma - u_{a} - \sigma^{s} $$

41 $$ \sigma^{s} = \left\{ \begin{gathered} - \left( {u_{{\text{a}}} - u_{{\text{w}}} } \right),u_{a} - u_{{\text{w}}} < 0 \hfill \\ - \frac{{\theta - \theta_{{\text{r}}} }}{{\theta_{{\text{s}}} - \theta_{{\text{r}}} }}\left( {u_{{\text{a}}} - u_{{\text{w}}} } \right),u_{{\text{a}}} - u_{{\text{w}}} > 0 \hfill \\ \end{gathered} \right. $$

where $\sigma^{^{\prime}}$ is the effective stress, *u*_w_ is the pore water pressure. According to the Mohr–Coulomb failure criterion, the shear strength of the sliding surface can be expressed as follows:

42 $$ \tau_{{\text{f}}} = c^{\prime} + \sigma ^{\prime}\tan \varphi ^{\prime} = c^{\prime} + \left[ {1 + \theta_{{{\text{zf}}}} + \frac{1}{2}\left( {\theta_{{\text{s}}} - \theta_{{{\text{zf}}}} } \right)\gamma_{{\text{d}}} z_{{\text{f}}} \cos \alpha - u_{{\text{a}}} - \sigma^{s} } \right]\tan \varphi ^{\prime} $$

*τ*_f_ is the shear strength, *c'* is the effective cohesion, and *φ'* is the effective internal friction angle. In the limit equilibrium state, the stability coefficient (*FOS*) of the slope is defined as the ratio of the sliding surface shear strength *τ*_f_ to the shear stress *τ*_s_, expressed as follows:

43 $$ FOS = \frac{{\tau_{{\text{f}}} }}{{\tau_{{\text{s}}} }} = \frac{c^{\prime} + \sigma ^{\prime}\tan \varphi ^{\prime}}{{1 + \theta_{{{\text{zf}}}} + \frac{1}{2}(\theta_{{\text{s}}} - \theta_{{{\text{zf}}}} )\gamma_{{\text{d}}} z_{{\text{f}}} \sin \alpha }} $$

*FOS* can be deformed as follows:

44 $$ FOS = A + B + C $$

45 $$ A = \frac{\tan \varphi ^{\prime}}{{\tan \alpha }} $$

46 $$ B = \frac{c^{\prime}}{{\left[ {1 + \theta_{{{\text{zf}}}} + \frac{1}{2}\left( {\theta_{{\text{s}}} - \theta_{{{\text{zf}}}} } \right)} \right]\gamma_{{\text{d}}} z_{{\text{f}}} \sin \alpha }} $$

47 $$ C = - \frac{{u_{a} + \sigma^{s} }}{{\left[ {1 + \theta_{{{\text{zf}}}} + \frac{1}{2}\left( {\theta_{{\text{s}}} - \theta_{{{\text{zf}}}} } \right)} \right]\gamma_{{\text{d}}} z_{{\text{f}}} \sin \alpha }}\tan \varphi ^{\prime} $$

## Calculation example verification

The numerical simulation results of Li, et al.^22^ are used to compare. The infiltration depth and *FOS* for the five rainfall durations of 6 h, 7 h, 8 h, 9 h, and 10 h are given by numerical simulation, where the rainfall intensity *q*_r_ = 4.333 × 10^–4^ m/min, the slope angle *α* = 30°, and the specific soil parameters are shown in Table 1.

**Table 1:** Basic parameters of soil.

VG model parameters					*ψ*/m	*γ*_d_/kN.m^-3^	*γ*_t_/kN.m^-3^	Unsaturated Soil Strength Parameters		
θ_r_/100%	θ_s_/100%	α/m^-1^	*n*	k_s_/ (m/min)				*φ'*	c*'*	*φ*^b^
1.5	40	3.5	1.5	3.47 × 10^–4^	0.06	13.57	19	25	3	30

**Figure 4:**
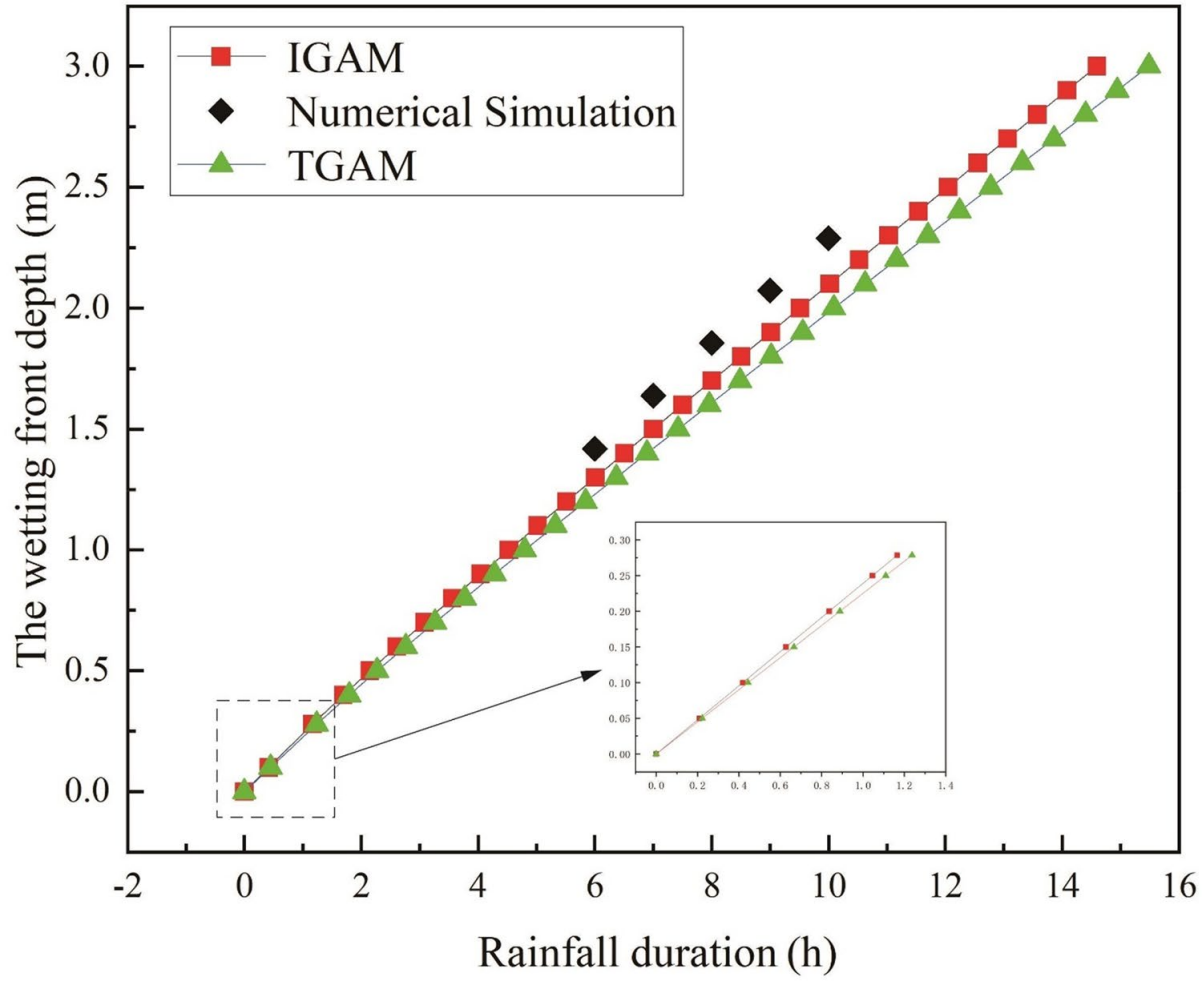
Variation of wetting front depth with rainfall time in different models.

In the figure, TGAM represents the traditional Green-Ampt model and IGAM represents the improved G-A model in this paper. It can be seen from Fig. 4 that in the early stage of rainfall infiltration, the boundary conditions of infiltration are controlled by the rainfall intensity, and the wetting front depth increases linearly with the rainfall time. The infiltration boundary of the soil is controlled by the soil’s infiltration capacity in the middle and later stages of rainfall. The infiltration rate gradually decreases and finally tends to be stable, and the depth of the wetting front shows a certain degree of nonlinearity with the rainfall time. With the infiltration time increasing, the infiltration model that does not consider the water distribution above the wetting front results in slower infiltration than the actual infiltration situation. The method proposed in this paper is closer to the actual infiltration situation. This is because it is assumed that the upper part of the wetting front is saturated, the moisture content of the soil is larger, and the wetting front reaches the same depth, which requires more infiltration time, and under the same rainfall conditions, longer rainfall infiltration times are required. Compared with the G-A model that does not consider the unsaturated region above the wetting front, the method proposed in this paper is more in line with the actual infiltration situation.

**Figure 5:**
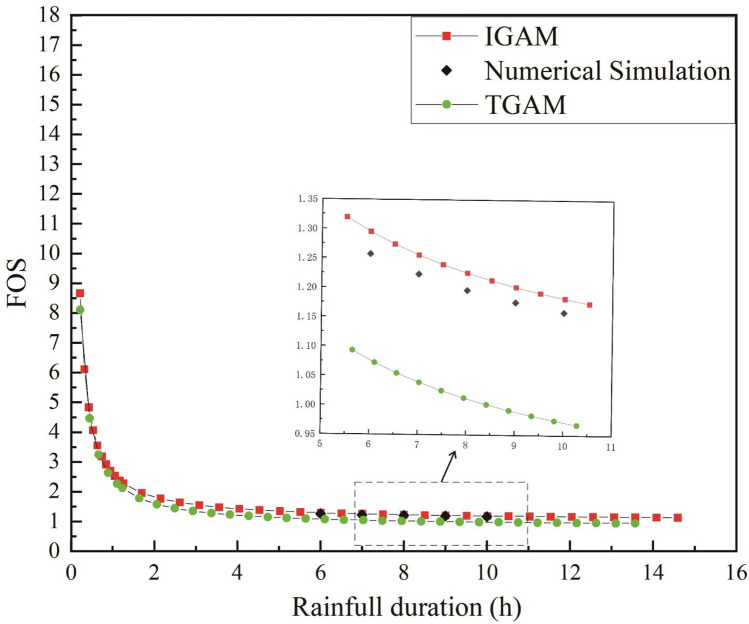
Variation of *FOS* of different models with rainfall duration.

**Figure 6:**
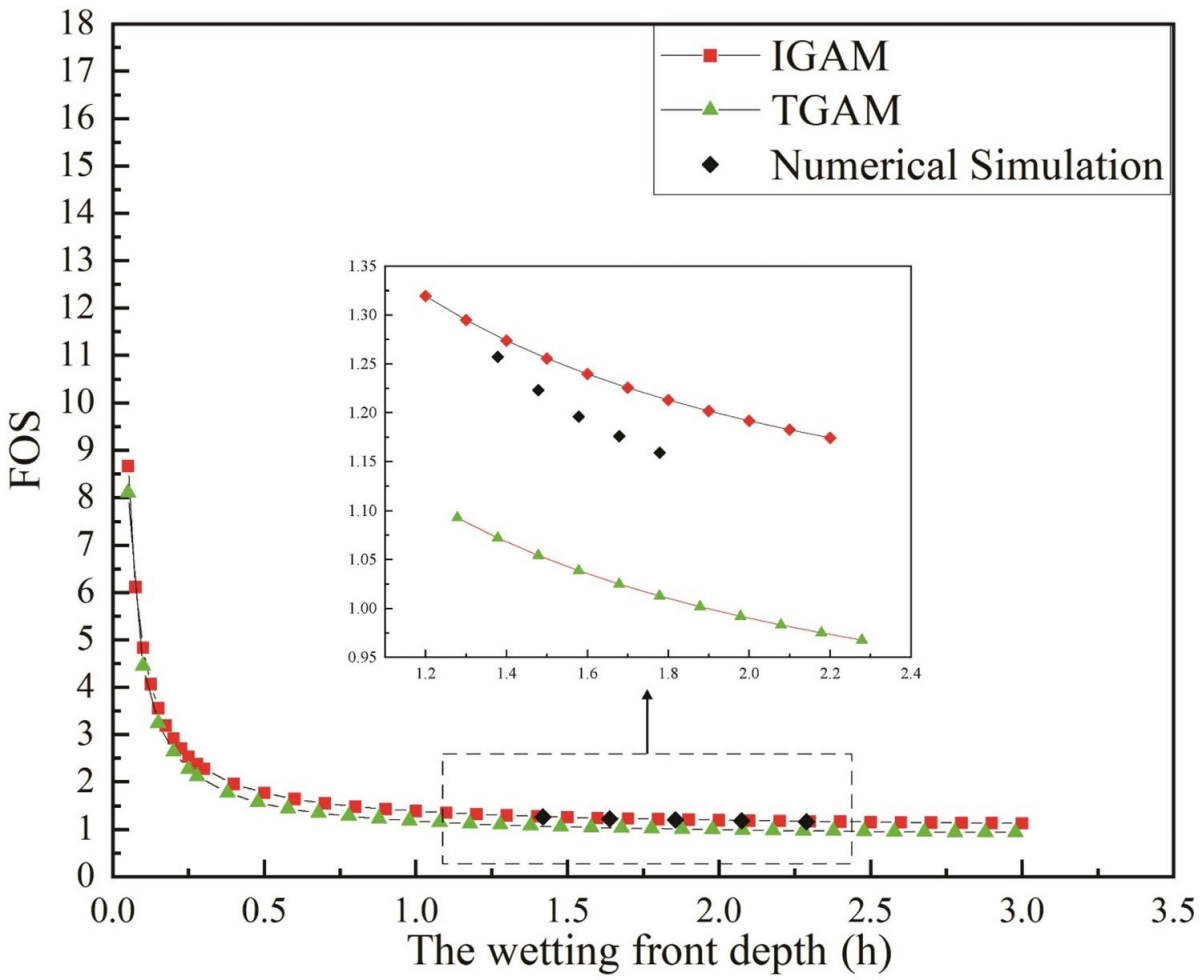
Variation of the *FOS* of different models with the depth of the wetting.

It can be seen from Figs. 5 and 6 that the *FOS* calculated by the two models show consistent regularity with time or infiltration depth. In the early stage of rainfall, the *FOS* calculated by the two models both decreased rapidly with the rainfall time. The rate of decrease of the stability coefficient calculated by the two models slows down with the continuous rainfall. Compared with the moisture content distribution without considering the wetting area, the *FOS* calculated by the method proposed in this paper is larger in the same rainfall infiltration time. This is because when the moisture content above the wetting front is considered to be the saturated moisture content. When calculating the *FOS* at the wetting front, the effect of matrix suction is often ignored. Therefore, the calculated *FOS* is often smaller than the actual value.

### The moisture content distribution of soil section

**Figure 7:**
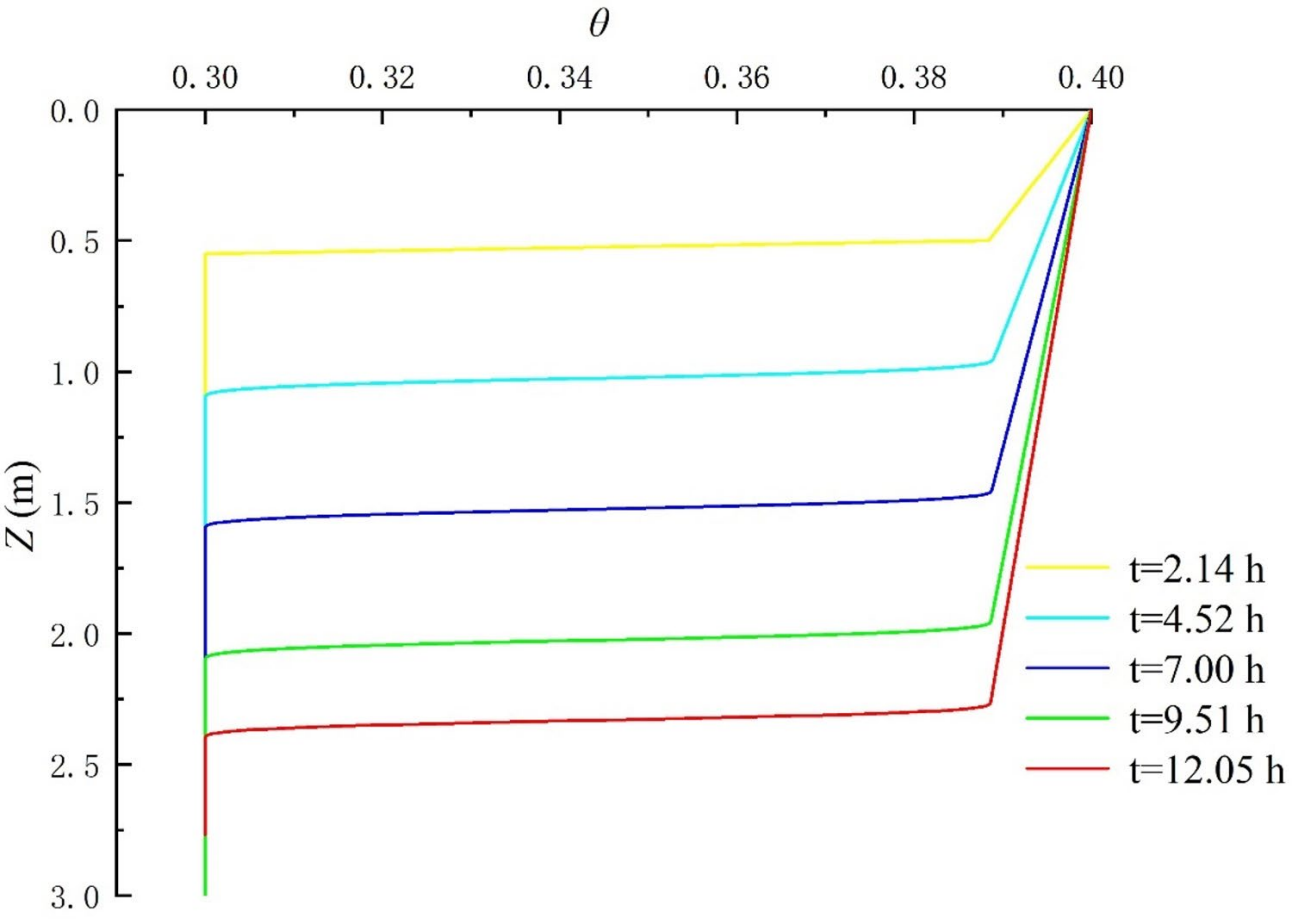
Schematic diagram of the variation of soil profile moisture content with rainfall.

The moisture content distribution in the soil section under different rainfall times are shown in Fig. 7. When the infiltration depths were respectively 0.5 m, 1.0 m, 1.5 m, 2.0 m, and 2.5 m, the required time was respectively 2.14 h, 4.52 h, 7.00 h, 9.51 h, and 12.05 h; the time needed for the wetting front to increase the depth of 0.5 m is 2.14 h, 2.38 h, 2.42 h, 2.51 h, and 2.54 h, respectively. It can be seen from the above infiltration law that as the depth of infiltration increases, the time required for infiltration increases; that is, the infiltration rate tends to decline.

### Influence of different rainfall intensities

**Figure 8:**
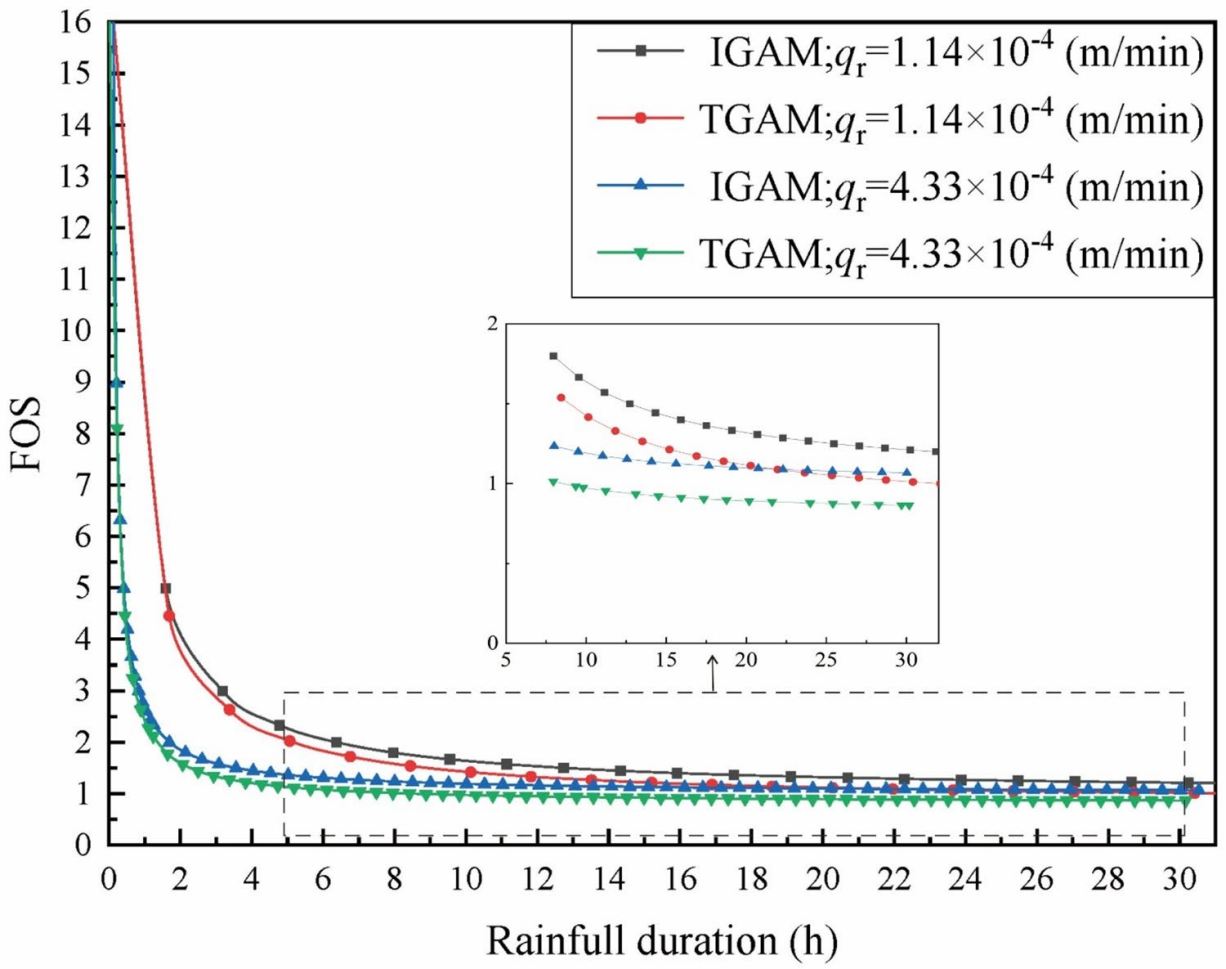
Variation of stability coefficient with rainfall time under different rainfall intensities.

**Figure 9:**
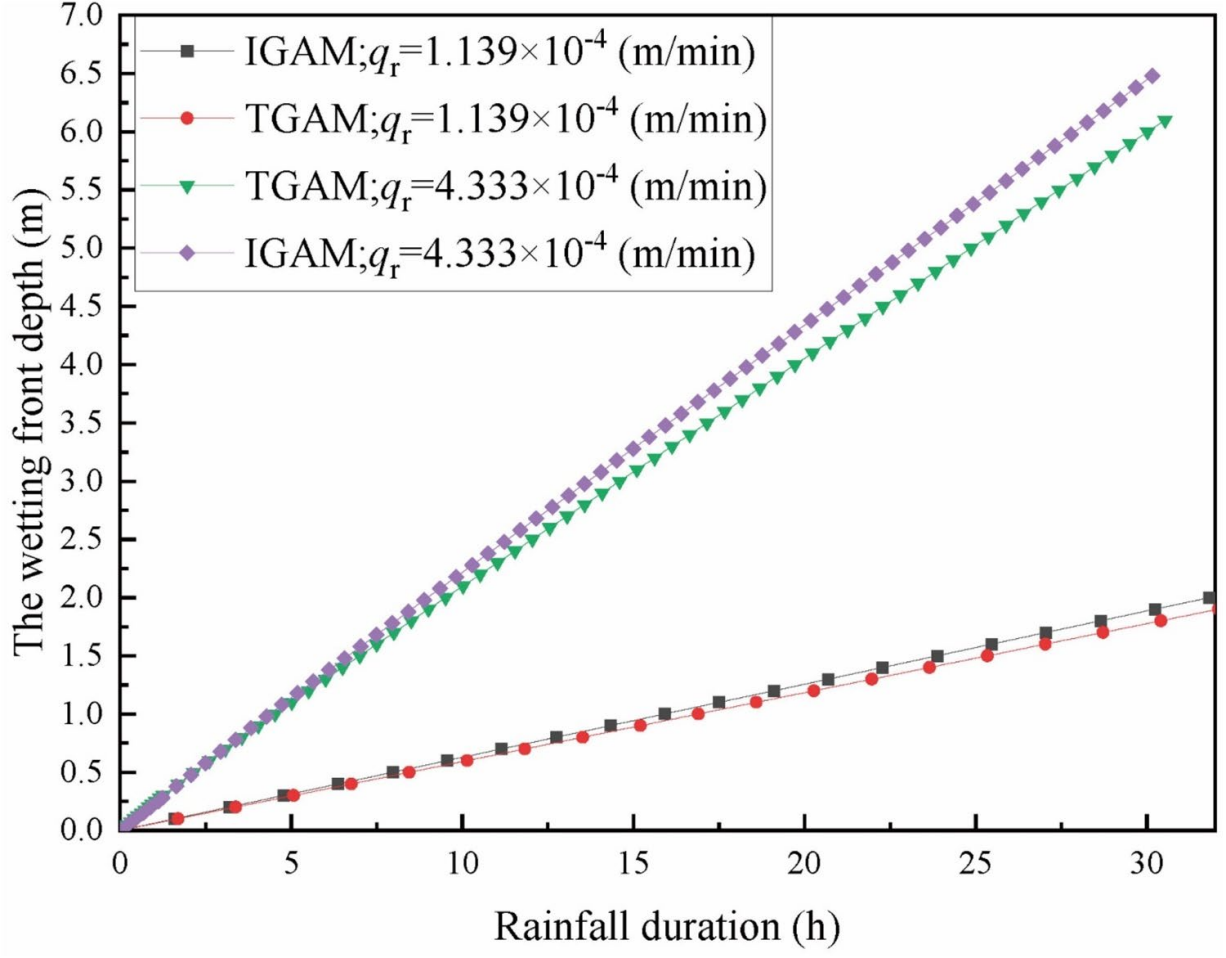
Variation of wetting front depth with rainfall time under different rainfall intensities.

The parameters of the soil are given in Table 1, the rainfall intensity *q*_r_ = 1.139 × 10^–4^ m/min is selected as the low-intensity rainfall condition, and the rainfall intensity *q*_r_ = 4.333 × 10^–4^ m/min is selected as the heavy rainfall condition. The variation of the wetting front with rainfall time and the variation of *FOS* with time under two rainfall conditions were calculated respectively. The results are shown in Figs. 8 and 9:

It can be seen from Fig. 8 that there is a great correlation between *FOS* and rainfall intensity. In the same rainfall duration, the decline of *FOS* under heavy rainfall is significantly faster than that under light rainfall. When the moisture content in the upper part of the wetting front is considered as a trapezoidal distribution, the decline of *FOS* is significantly lower than that of the traditional G-A model. It is worth noting that when the rainfall duration is more than 21 h. The *FOS* calculated by the traditional G-A model under light rainfall conditions is smaller than that of the assumption that the upper part of the wetting front is trapezoidal moisture content under heavy rainfall conditions. It can be seen from Fig. 9 that the infiltration considering the water distribution in the upper part of the wetting front is faster than that of the traditional G-A model, and this phenomenon is more obvious when the rainfall intensity is larger. Therefore, in the same rainfall duration, the moisture content in the upper part of the wetting front is equivalent to a trapezoidal distribution, although the infiltration is deeper, the calculated *FOS* is greater.

### Influence of different slopes

**Figure 10:**
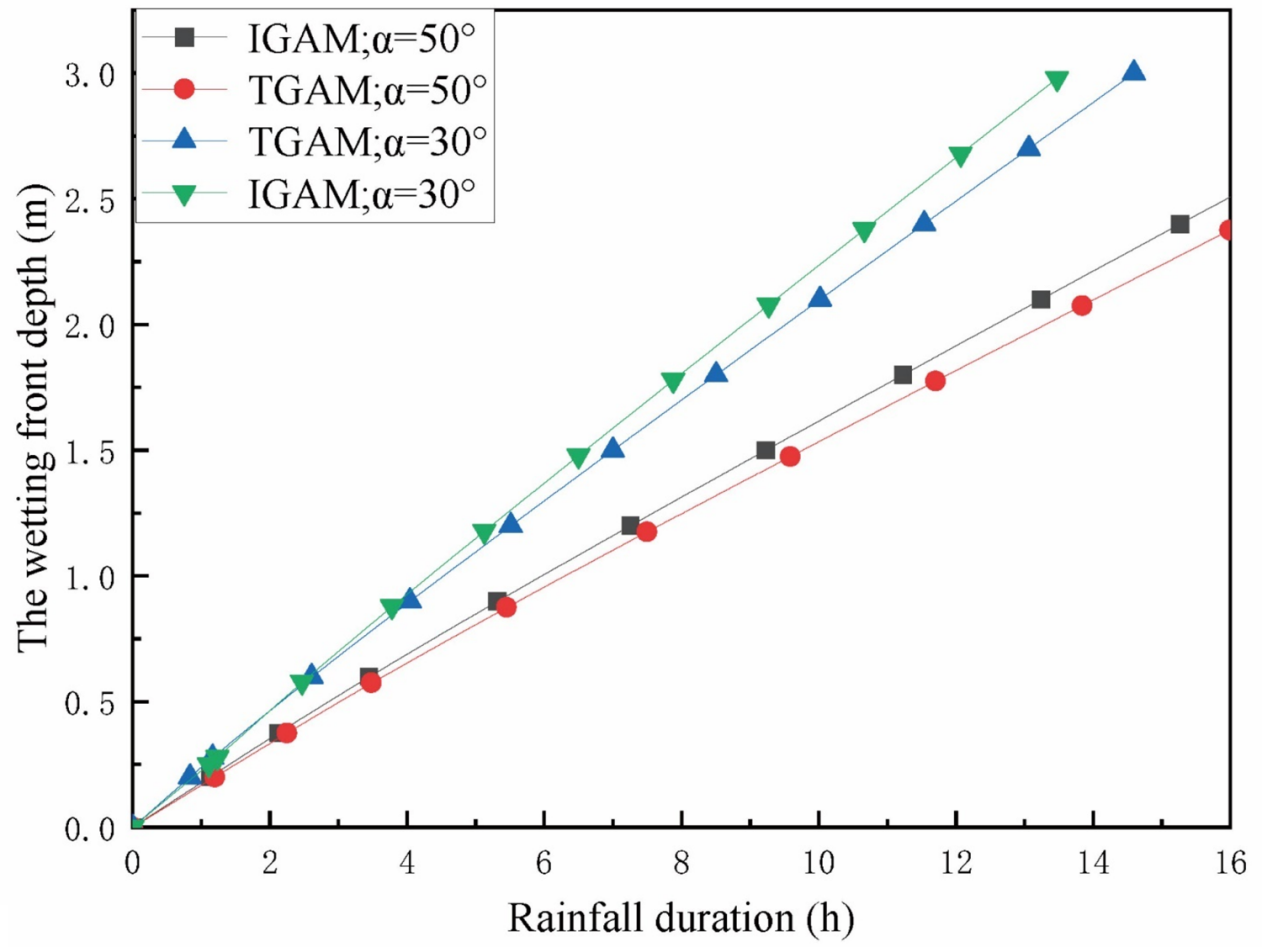
Variation of wetting front depth with rainfall time at different angles.

**Figure 11:**
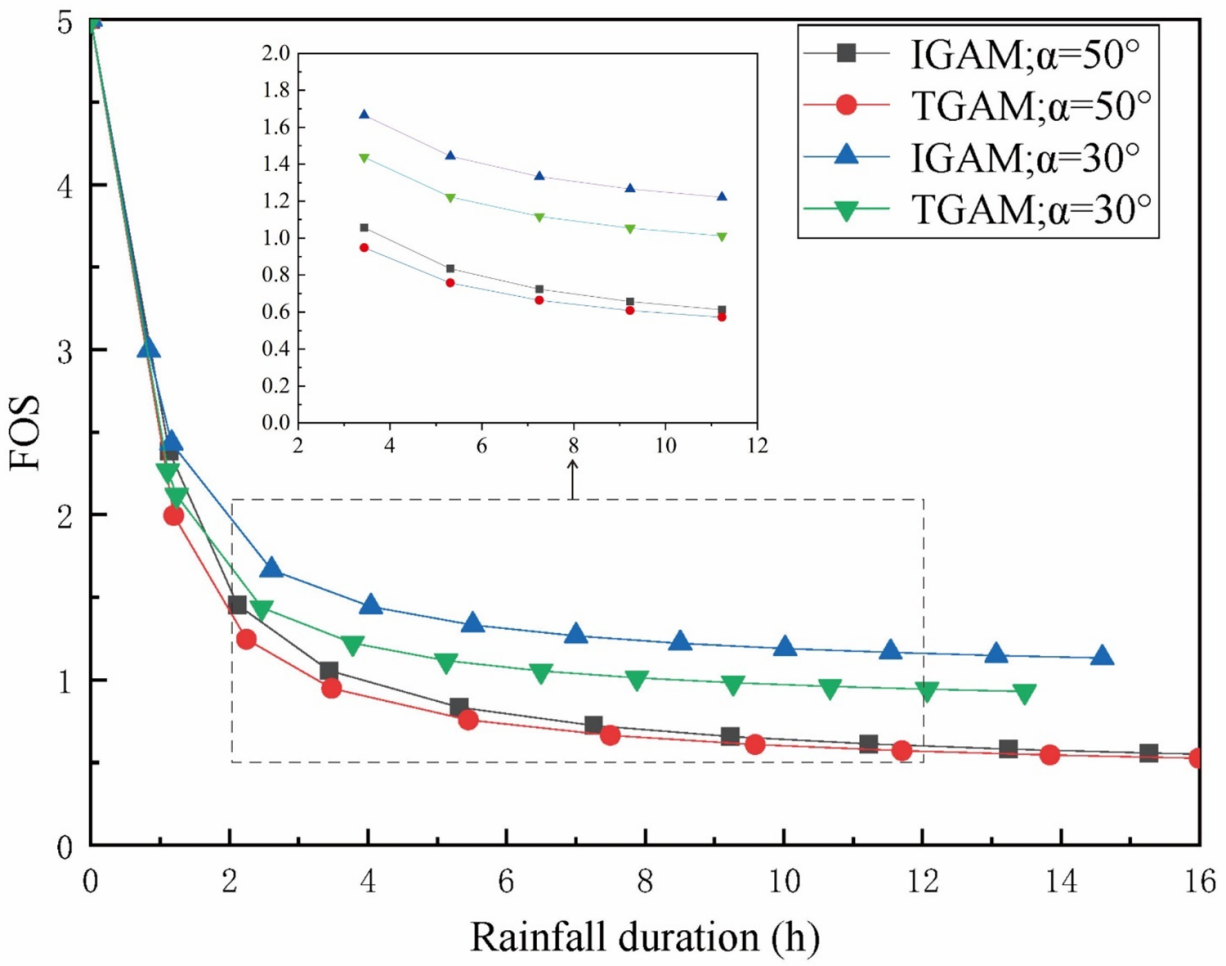
Variation of *FOS* with rainfall time at different angles.

In order to study the variation law of slope angle on infiltration and slope stability. The slope angles of 30° and 50° were selected respectively, and the variation of infiltration depth and *FOS* with rainfall time was calculated. The results are shown in Figs. 10 and 11.

It can be seen from Fig. 10 that with the increase of the slope angle, the wetting front depth decreases in the same rainfall duration. This is because the angle between the rain and the slope decreases as the slope angle increases, the infiltration in the direction perpendicular to the slope surface is reduced making infiltration more difficult. It can be seen from Fig. 11 that the larger the slope angle, the worse the slope stability during the same rainfall time. It is worth noting that the upper part of the equivalent wetting front is equivalent to trapezoid and the traditional G-A model method the *FOS* calculated is more different, when the slope changes from 30° to 50°. It can be seen from Eq. (42) that when the soil parameters are determined, the *FOS* of the slope only depends on the depth and slope angle of the wetting front, and the increase of the slope angle will reduce the depth of the wetting front and the slope stability at the same time. It shows that the slope angle has a significant effect on the strength of the slope during the whole process of rainfall infiltration.

### Variable-rate rainfall intensity

It is obvious that the rainfall intensity cannot be constant. The analysis model of variable rain intensity was proposed by Chu through improved G-A model^11^. Assuming that *R*_i-1_ *R*_i_ is the rainfall at *t*_i-1_ *t*_i_ time, *I*_i-1_, *I*_i_ corresponds to the cumulative infiltration at *t*_i-1_ *t*_i_ time, and *q*_r_ is the rainfall intensity at *t*_i_ time, the whole rainfall process can be divided into four cases.

Case 1, when there is no ponding in the slope at the beginning, there is no ponding in the slope at the end, and the rainfall intensity in this case is always less than the infiltration intensity. The judgment equation is as follows:

48 $$ C{\text{u = R}}_{{\text{i}}} - (R_{{\text{i - 1}}} - I_{{\text{i - 1}}} ) - K_{{\text{s}}} \frac{{h_{{\text{f}}} \left[ {\frac{1}{2}(\theta_{{{\text{zf}}}} + \theta_{{\text{s}}} ) - \theta_{{\text{i}}} } \right]}}{{q_{{\text{r}}} \cos \alpha - K_{{\text{s}}} }} < 0 $$

The relationship between the depth of wetting front and time in the whole stage is as follows:

49 $$ z_{{\text{f}}} = \frac{{\int_{{t_{n - 1} }}^{{t_{n} }} {{\text{q}}_{{\text{r}}} {(}t)\cos \theta dt} }}{{\left[ {\left( {\theta_{{z{\text{f}}}} - \theta_{i} } \right) + \frac{1}{2}\left( {\theta_{s} - \theta_{{z{\text{f}}}} } \right)} \right]}} $$

Case 2, the stage of *t*_n-1_ without ponding and *t*_n_ with ponding is divided into two parts, the rainfall control stage and the slope control stage. The judgment formula is as follows:

50 $$ C{\text{u = R}}_{{\text{i}}} - (R_{{\text{i - 1}}} - I_{{\text{i - 1}}} ) - K_{{\text{s}}} \frac{{h_{{\text{f}}} \left[ {\frac{1}{2}(\theta_{{{\text{zf}}}} + \theta_{{\text{s}}} ) - \theta_{{\text{i}}} } \right]}}{{q_{{\text{r}}} \cos \alpha - K_{{\text{s}}} }} > 0 $$

The critical time *t*_x_ of rainfall control phase and slope control phase can be calculated by the following:

51 $$ t_{x} = \frac{{I_{p} \left( {t_{x} - t_{n - 1} } \right)}}{{\int_{{t_{n - 1} }}^{{t_{x} }} {q(t)\cos \alpha dt} }} = \frac{{\left[ {\left( {\theta_{{z{\text{f}}}} - \theta_{i} } \right)z_{p} + \frac{1}{2}\left( {\theta_{s} - \theta_{{z{\text{f}}}} } \right)z_{p} } \right]\left( {t_{x} - t_{n - 1} } \right)}}{{\int_{{t_{n - 1} }}^{{t_{x} }} {q(t)\cos \alpha dt} }} $$

Then the relationship between infiltration depth *z*_f_ and time *t* in the two stages of rainfall control and slope control is as follows:

52 $$ \frac{{z_{f} }}{t} = \left\{ \begin{gathered} \frac{{\int_{{t_{n - 1} }}^{{t_{x} }} {q(t)\cos \alpha dt} }}{{\left[ {\left( {\theta_{{z{\text{f}}}} - \theta_{i} } \right) + \frac{1}{2}\left( {\theta_{s} - \theta_{{z{\text{f}}}} } \right)} \right]\left( {t_{x} - t_{n - 1} } \right)}},t \le t_{x} \hfill \\ \frac{{K_{s} (\cos \alpha + \frac{{h_{{\text{f}}} }}{{z_{{\text{f}}} }})}}{{\left( {\theta_{{z{\text{f}}}} - \theta_{i} } \right) + \frac{1}{2}\left( {\theta_{s} - \theta_{{z{\text{f}}}} } \right)}},t \ge t_{x} \hfill \\ \end{gathered} \right. $$

Case 3, there is ponding at the beginning and ponding at the end, and the whole infiltration process is controlled by the slope itself and has nothing to do with the intensity of rainfall. The judgment equation is as follows:

53 $$ C_{p} = R_{i} - (I_{i} - I_{i - 1} ) - \left( {R_{i - 1} - I_{i - 1} } \right) > 0 $$

The relationship between infiltration depth *z*_f_ and time *t* at this stage is as follows.

54 $$ \frac{{dz_{{\text{f}}} }}{dt} = \frac{{K_{s} (\cos \alpha + \frac{{h_{{\text{f}}} }}{{z_{{\text{f}}} }})}}{{\left( {\theta_{{z{\text{f}}}} - \theta_{i} } \right) + \frac{1}{2}\left( {\theta_{s} - \theta_{{z{\text{f}}}} } \right)}},t_{x} \le t \le t_{n} $$

Case 4, when there is ponding in *t*_n-1_, there is no ponding in *t*_n_, and the rainfall intensity of this process is less than the infiltration rate. The judgment equation is as follows:

55 $$ C_{p} = R_{i} - (I_{i} - I_{i - 1} ) - \left( {R_{i - 1} - I_{i - 1} } \right) < 0 $$

When the rainfall time is short, net infiltration will no longer occur in this process. The infiltration in this process is ignored.

## Discussion

**Figure 12:**
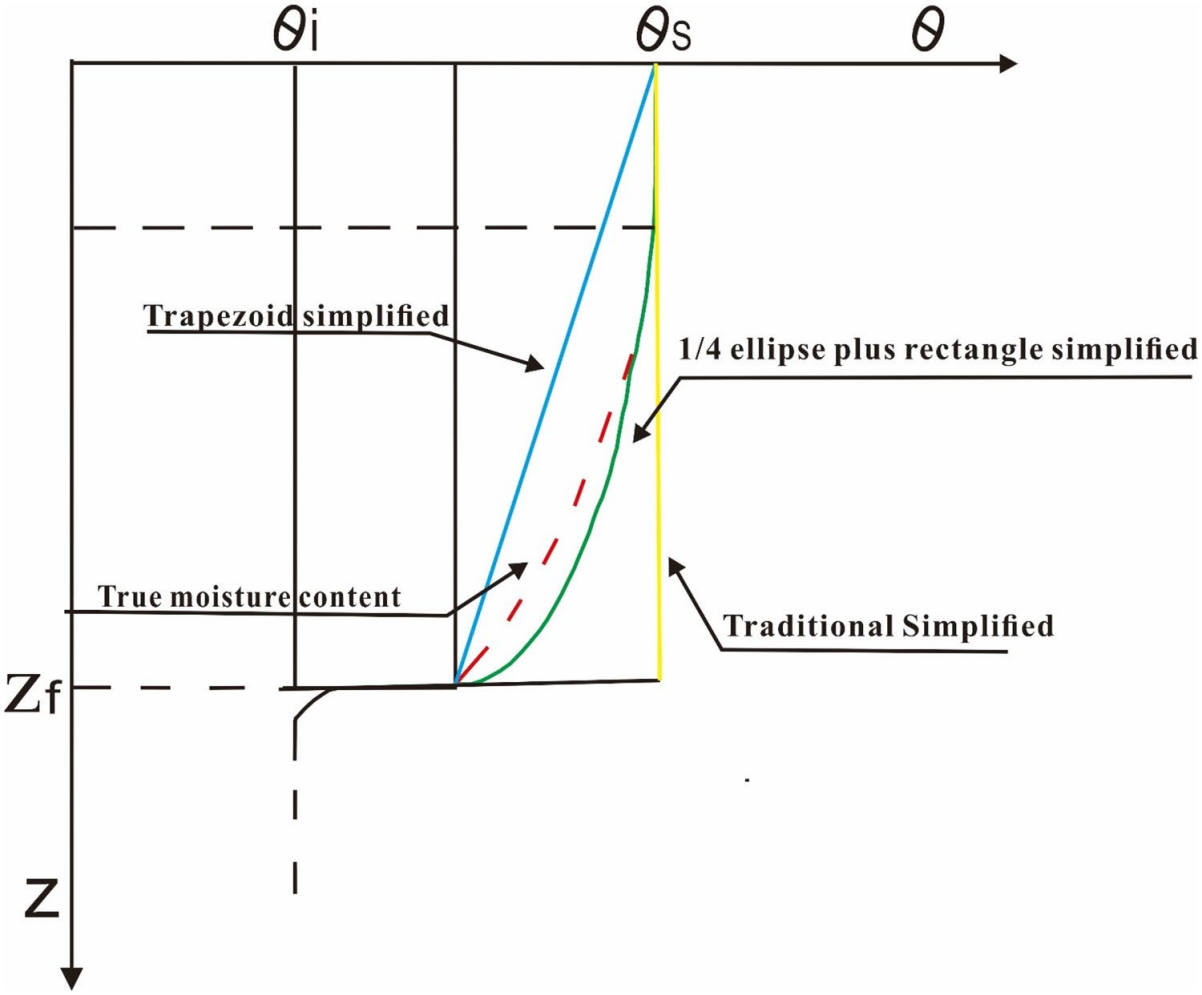
Comparison of simplified models for different water distribution.

In the actual infiltration situation, during the rainfall infiltration process, the soil will present three parts (i.e., the saturated area transition area and natural moisture content area). The cumulative infiltration and infiltration rate of 1/4 oval and rectangular combined model and trapezoidal model were compared by Zhang, et al.^18^ experimentally with the real infiltration. The results are shown in Fig. 12. The result shows that at the beginning of the rainfall, the equivalent trapezoid results are better than the traditional G-A model but slightly worse than the 1/4 ellipse and rectangle combination model. As the duration of the rainfall increases, the trapezoid equivalent is much better than that of combination model of rectangle and 1/4 ellipse and traditional G-A infiltration model. This is because as the duration of the rainfall increases, the soil moisture content at the equivalent 1/4 ellipse is much larger than the actual soil moisture content. However, in the trapezoidal equivalent moisture content model, the bottom of the wetting front is higher than the actual moisture content and the top of the wetting front is lower than the actual moisture content, the moisture content of top of the wetting front and the moisture content of bottom of the wetting front cancel each other out to a certain extent, making the trapezoidal equivalent total moisture content more in line with the actual situation. For the estimation of agricultural irrigation depths with short infiltration times, the rectangle and 1/4 trapezoid combination model is preferable. However, the slope calculation *FOS* is generally long in the selection of rainfall time. Therefore, the trapezoidal equivalent method is more suitable for slope *FOS* calculation.

**Figure 13:**
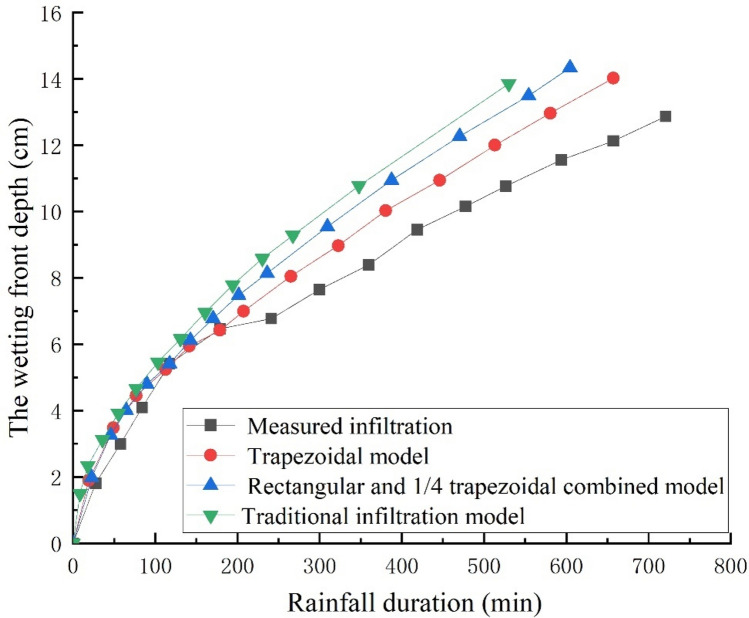
Cumulative infiltration depth and measured infiltration.

It is worth noting that, although the trapezoidal simplified model of moisture content in the upper part of the wetting front proposed in this paper is more accurate in accumulative infiltration than the 1/4 ellipse and rectangle combination model and the traditional G-A model. The cumulative infiltration is shown in Fig. 13. But compared with the real infiltration situation, the equivalent method proposed in this paper still has the disadvantage of higher cumulative infiltration. Due to the real infiltration is very complicated. The compression of the gas in the soil will produce an air entrapment effect^1^, the compaction of the soil will become denser with the depth, and the expansive soil particles in the soil will expand and compress the soil pores under the action of rainwater^23^, the wetting front is not simple, as there is the finger instability phenomenon^24^. All these will lead to the deviation between the cumulative infiltration in the actual situation and the actual situation. The effects of these aspects on infiltration have been considered. However, how to combine these effectively to get a more accurate infiltration model is the next step should be considered.

## Conclusion

In this paper, the moisture content at the wetting front is calculated, based on Darcy's law and the V-G model of unsaturated soil, then the moisture content distribution in the upper part of the wetting front is simplified to a trapezoid. Combined with the slope G-A infiltration model, a rainfall infiltration model considering the change of moisture content in the wetting area of the slope with depth is deduced. Combined with the G-A equation of infinite rainfall-induced slope infiltration and the shear strength theory of unsaturated soil, the calculation formula of the *FOS* of rainfall-induced landslide considering the distribution of soil water above the wetting front is proposed. And applying it to the stability analysis of infinite slopes under rainfall conditions. The following conclusions are obtained:

1. Through the analysis of the analytical expression form, the analytical expression of the *FOS* of rainfall-induced landslide considering the water distribution in the upper part of the wetting front is mainly composed of three parts. The factors affecting the stability of the slope under the action of rainfall can be summarized as slope angle, soil mechanics parameters and effects of matrix suction in unsaturated soils.
2. Compared with the saturated moisture content and combined model of 1/4 ellipse and trapezoidal distribution in the upper part of the wetting front. When the soil moisture content in the upper part of the wetting front is considered to be trapezoidal, the rainfall infiltration of the wetting front on the slope is faster, but the cumulative infiltration is smaller.
3. When the moisture distribution above the wetting front is considered to be trapezoidal, the calculated *FOS* of the slope increases significantly, especially when the slope angle is large and the heavy rainfall

## Data Availability

All data generated or analyzed during this study are included in the paper.
